# Editorial: New insights into extracellular vesicles in cardiovascular disease: Molecular basis, diagnosis and therapy

**DOI:** 10.3389/fcell.2022.989150

**Published:** 2022-08-25

**Authors:** Yang Shen, Xiaoheng Liu

**Affiliations:** Institute of Biomedical Engineering, West China School of Basic Medical Sciences & Forensic Medicine, Sichuan University, Chengdu, China

**Keywords:** extracellular vesicles, matrix vesicles, cardiovascular disease, cell and molecular biology, bioengineering

Extracellular vesicles (EVs) are a heterogeneous group of membranous structures released by cells under physiological and pathological conditions (Guillaume et al., 2018). According to the density, size and subcellular origin, EVs are generally divided into three categories: apoptotic body (100–5000 nm), microvesicles (100–1000 nm), and exosomes (50–150 nm) (Mathieu et al., 2019). Noteworthy, Matrix vesicles (MVs, 100–300 nm) are now considered a specific type of EVs secreted by mineral-forming cells (Kirsi et al., 2019). So far, EVs attract widespread attention due to their diverse roles in intercellular and intracellular communication (Graça and Stahl, 2019; Guillaume et al., 2022). EVs are associated with immune responses, viral pathogenicity, cardiovascular diseases and cancer progression (Cheng and Hill, 2022). In cardiovascular research, EVs present a promising therapeutic strategy for the treatment of cardiovascular diseases or blockage of disease progression (Susmita et al., 2021). The Research Topic “*New insights into extracellular vesicles in cardiovascular disease: Molecular basis, diagnosis and therapy*” of Frontiers in Cell and Developmental Biology aims to pick exciting and noteworthy works related to the mechanistic investigation of EVs and their translational applications in cardiovascular disease. Collectively, this Research Topic comprises three original research articles and six review articles for a total of nine original contributions, articulating the roles of EVs in atherosclerosis, vascular calcification, and other cardiovascular diseases. These articles not only highlight the molecular basis, diagnosis and therapeutic potential of EVs, but reveal a field of research moving forward at an incredible pace.

This Research Topic includes three original research articles demonstrated the role and mechanism of EVs in cardiovascular disease. Liu et al. investigated the contribution of platelet-derived microvesicles (PMVs) in phenotypic switch of vascular smooth muscle cells (VSMCs) during vascular remodeling. This work revealed that PMVs secreted by activated platelets promoted VSMC dedifferentiation *via* Src/Lamtor1/mTORC1 signaling pathway, suggesting that Lamtor1 may be a potential therapeutic target for intimal hyperplasia after injury. The study by Yaker et al. showed that EVs derived from lipopolysaccharide-treated macrophages can aggravate vascular calcification through activation of pro-inflammatory and pro-oxidative responses in VSMCs. Comariţa et al. found the therapeutic potentiality of stem cell-derived EVs secreted from subcutaneous adipose tissue stem cells (ADSCs) and bone marrow mesenchymal stem cells (MSCs) on atherosclerosis-induced vascular dysfunction. Stem cell-derived EVs were identified contributing to the regression of atherosclerosis-induced endothelial dysfunction by improving the structure and function of the vascular wall. In combination with Smad2/3siRNA, the ability of ADSCs or MSCs-derived EVs was significantly amplified to regress the inflammation-mediated atherosclerotic process.

Additionally, six comprehensive review articles discussed the multifaceted functions of EVs in cardiovascular health. EVs act as extracellular biological information carriers that transfer a variety of functional transcripts and lipids to target cells to mediate cell-cell communication. Moreover, the inherent properties of EVs have great potentials to be used as biomarkers for diagnosis and prognosis of cardiovascular disease, and to play a therapeutic role as a drug or drug carrier.

Martin-Ventura et al. reviewed the role of EVs as potential diagnostic and prognostic biomarkers in chronic cardiovascular diseases, including atherosclerosis, aortic stenosis and aortic aneurysms. And they further summarized the pathological mechanisms of EVs-mediated vascular and valvular calcification, involving calcium accumulation and osteogenic phenotypic transition of VSMCs or valvular interstitial cells. Similarly, MVs are especially critical in extracellular matrix mineralization and the development of vascular calcification (Kapustin et al., 2011). Li et al. discussed the detailed roles of MVs in the regulation of vascular calcification, the possible mechanism of which involves mineral deposits, osteogenic transdifferentiation of VSMCs, and microRNAs transport.

Exosomes are small EVs (30–150 nm) coated with bi-lipid membranes and contain numerous bioactive molecules (Kalluri and LeBleu, 2020). Germena et al. summarized the current knowledge on the role of exosomes as mediators of cardiovascular diseases in atherosclerosis and diabetes. Moreover, they described evidence of intercellular connection among multiple cell types (cardiac, vasculature, immune cells) as well as the challenge of their *in vivo* analysis. Burtenshaw et al. generalized exosomal composition, biogenesis and profiling, and discussed how key exosomal signatures in liquid biopsies may act as early pathological indicators of adaptive lesion formation and arteriosclerotic disease progression.

The review by Wen et al. discussed the great potential of EV-derived circular RNAs (EV-circRNAs) as diagnostic biomarkers of atherosclerosis. And they depicted the mechanism of EV-circRNAs in regulating atherosclerosis formation and development, including EC dysfunction, phenotypic switching of VSMCs, inflammatory response, lipid deposition, and formation of foam cells. Thus, EV-circRNAs are expected to be used as novel therapeutic strategies for atherosclerosis.

Recent studies have highlighted that excess activation of NLRP3 inflammasome led to inflammation and the progression of atherosclerosis (Sharma and Kanneganti, 2021). Lu et al. reviewed the recently described mechanisms of the NLRP3 inflammasome activation, and discuss emphatically the pharmacological interventions using statins and natural medication for atherosclerosis associated with NLRP3 inflammasome. Thus, continuously developing the specific NLRP3 inhibitors may be promising therapeutic remedies to solve atherogenesis.

In summary, this Research Topic covers the broadest possible aspects of EVs and highlights the vital role of EVs in cardiovascular diseases. Based on the research findings and currently known key points, we hope to make a further understanding and better characterization of this promising but not yet well-understood field.

The success of this Research Topic is the joint efforts of everyone. We would like to thank all the authors for their contribution to the Research Topic. At the same time, we would like to appreciate the efforts of all the reviewers and editors.
